# Genome-Wide Association Studies in Indian Buffalo Revealed Genomic Regions for Lactation and Fertility

**DOI:** 10.3389/fgene.2021.696109

**Published:** 2021-09-20

**Authors:** Vikas Vohra, Supriya Chhotaray, Gopal Gowane, Rani Alex, Anupama Mukherjee, Archana Verma, Sitangsu Mohan Deb

**Affiliations:** Buffalo Breeding Lab, Animal Genetics and Breeding Division, Indian Council of Agricultural Research-National Dairy Research Institute, Karnal, India

**Keywords:** Murrah, buffaloes, GWAS, lactation persistency, milk yield, fertility

## Abstract

Murrah breed of buffalo is an excellent dairy germplasm known for its superior milk quality in terms of milk fat and solids-not-fat (SNF); however, it is often reported that Indian buffaloes had lower lactation and fertility potential compared to the non-native cattle of the country. Recent techniques, particularly the genome-wide association studies (GWAS), to identify genomic variations associated with lactation and fertility traits offer prospects for systematic improvement of buffalo. DNA samples were sequenced using the double-digestion restriction-associated DNA (RAD) tag genotyping-by-sequencing. The bioinformatics pipeline was standardized to call the variants, and single-nucleotide polymorphisms (SNPs) qualifying the stringent quality check measures were retained for GWAS. Over 38,000 SNPs were used to perform GWAS on the first two principal components of test-day records of milk yields, fat percentages, and SNF percentages, separately. GWAS was also performed on 305 days’ milk yield; lactation persistency was estimated through the rate of decline after attaining the peak yield method, along with three other standard methods; and breeding efficiency, post-partum breeding interval, and age at sexual maturity were considered fertility traits. Significant association of SNPs was observed for the first principal component, explaining the maximum proportion of variation in milk yield. Furthermore, some potential genomic regions were identified to have a potential role in regulating milk yield and fertility in Murrah. Identification of such genomic regions shall help in carrying out an early selection of high-yielding persistent Murrah buffaloes and, in the long run, would be helpful in shaping their future genetic improvement programs.

## Introduction

Buffalo (*Bubalus bubalis*) is an imperative livestock species and act as a key component for improving agricultural economy and supplying milk, meat, and draft power. The buffalo population across the world was recently estimated to be 194 million, 97% of which were present in Asia (FAO, 2014). Buffalo is well known for its high milk quality, with higher fat (6.4–8.0% vs. 4.1–5.0%) and protein (4.0–4.5% vs. 3.4–3.6%) contents than cow milk (Venturini et al., 2014; Khedkar et al., 2016). The concentration of these milk constituents offers a higher economic return of the buffalo milk and increases the demand of value-added products like mozzarella. Buffaloes are an integral and crucial genetic resource in Indian dairy industry, contributing 49% to the total milk produced according to the basic animal husbandry statistics, 2019^1^. About 63% of global buffalo milk production and 95% of Asian buffalo milk production is contributed by Indian buffaloes (FAO, 2014). Buffaloes are more adaptable to harsh environments and often resist various bovine tropical diseases. However, the poor reproductive efficiency of buffaloes limits its potential. Buffaloes exhibit higher age at puberty and maturity, longer postpartum breeding interval, and low conception rates (Gordon, 1996; Warriach et al., 2015; Raina et al., 2016). Buffaloes in the field condition also suffer from short lactation of 252–270 days as compared to the standard 300 days (Ganguli, 1981; Griffiths, 2010). This underlines the scope for improving the milk production and fertility potential through implementation of systematic breeding programs for buffaloes in the country. However, in India, very few works have been done in order to identify the infinitesimally large number of underlying loci regulating the expression of complex traits such as milk production, lactation persistency and fertility.

Genome wide association studies (GWAS) in buffaloes for lactation traits have been mainly limited to the use of bovine single-nucleotide polymorphism (SNP) chip (Wu et al., 2013; Venturini et al., 2014) and Affymetrix’s buffalo 90K SNP chip (de Camargo et al., 2015; Iamartino et al., 2017; Liu et al., 2018). There have been very few GWAS conducted in India covering all aspects of production and reproduction performance due to constraints of cost incurred and organized large-scale genotyping programs. High-density SNP panels are a prerequisite for GWAS, which have led to developments of cost-effective and efficient next-generation sequencing (NGS) technologies such as reduced representation of genomic libraries (RRLs) (Van Tassell et al., 2008). The flexibility, robustness, and low cost of double-digestion restriction-associated DNA (RAD) tag genotyping-by-sequencing (ddRAD-GBS) technique renders it suitable for identification of SNPs in any species for GWAS (Davey et al., 2011; Elshire et al., 2011).

Hence, the present study was conducted to identify novel SNPs associated with milk production, composition, lactation persistency, and fertility traits at the genomic level using the genotype-by-sequencing technique in Murrah buffalo, India’s major buffalo breed and milch animal of the nation.

## Materials and Methods

### Sampling, Data Recording, and Genotyping

A total of 672 test-day records on each trait, i.e., milk yield (TDMY), fat percentage (TDFP), and solids-not-fat (SNF) percentage (TDSNF) were collected from 96 female Murrah buffaloes reared at LRC, NDRI, Karnal, India (29.68°N and 76.99°E). Records of 96 buffaloes on 305 days’ milk yield (305DMY), birth weight (bwt), age at first calving (AFC), calving interval (CI), and age at sexual maturity (ASM) in months were collected. Other traits such as lactation persistency, postpartum breeding interval in days (PPBI), and breeding efficiency (BE) were derived from the primary phenotype records. Breeding efficiency was calculated for female buffaloes as described by Tomar (1965).

Lactation persistency was estimated by following four different methods:

(I) Wood (1967) incomplete gamma function, where individuals were classified as persistent and non-persistent as described by Macciotta et al. (2005) considering a positive rate of incline as a favorable condition and a negative rate as an unfavorable condition for persistency
(II) Johansson and Hansson (1940) method
(III) Ludwick and Petersen (1943) method
(IV) Mahadevan (1951) method

DNA was isolated from 96 Murrah buffaloes selected for the study following the standard phenol-chloroform method (Sambrook and Russell, 2006). Samples were further processed using the standard RAD protocol as described by Peterson et al. (2012). DNA double digestion was carried out with *Sph*I and *Mlu*CI restriction enzymes. Adapters (P1 and P2) were prepared as per standard Illumina read multiplexing protocol using an inline barcode along with Illumina index for library preparation. After adapter ligation and size selection, samples were sequenced on a Illumina Hi-Seq 2000 platform.

### Variant Calling

The NGS pipeline was standardized after incorporating a few modifications in the standard mpileup variant calling pipeline (Li, 2011), to call variants present in the Murrah population. The Mediterranean buffalo genome, having accession ID GCF_003121395.1, was retrieved from the NCBI dataset and used as a reference genome. Index and sequence dictionary files were created using the Burrows–Wheeler algorithm (BWA) (Li and Durbin, 2010) and PicardTools,^2^ respectively. The quality of paired-end raw FASTQ files generated after sequencing was checked using FastQC (Andrews, 2010), and each report was combined through MultiQC (Ewels et al., 2016). Adapters were marked and trimmed using bbmap (Brian, 2014).^3^ The BWA-MEM algorithm was used to align the trimmed FASTQ sequences with the reference genome. Aligned files were coordinate-sorted, and duplicate reads were removed. Read group identifiers were updated using PicardTools. The quality of aligned BAM files was checked using qualimap (García-Alcalde et al., 2012). Variants were called using bcftools-mpileup (Li, 2011).

### Quality Control of Variants

Only biallelic SNPs having more than 95% genotyping rate were retained for further GWAS. SNPs with a MAF < 0.05 and deviating from the Hardy–Weinberg equilibrium at *p* < 0.0001 were removed from the dataset. SNPs in LD with *r*^2^ > 0.8 were also removed. Only autosomal and X chromosome SNPs were retained for the final analysis. All the quality control operations and data preprocessing were performed using PLINK v1.9 (Chang et al., 2015).

### Statistical Model

GWAS for milk yield, fat percentage, and SNF percentage were conducted on the principal components (PCs) instead of direct traits. Principal component analysis (PCA) was performed in the R programming environment (v4.0.3) on the 672 test-day records of each trait separately. As the first two PCs cumulatively account for most of the variation in the dataset, they were selected to be GWAS traits. The traits on which GWAS were performed were PCs (PC_1_ and PC_2_) of TDMYs, TDFPs, TDSNFs, and 305DMY; lactation persistency calculated by four different methods; age at sexual maturity in months; postpartum breeding interval (in days); and breeding efficiency. Genome-wide identity-by-state (IBS) for all pairs of individuals was checked. Multidimensional scaling (MDS) based on SNP information was done to check for the presence of any population stratification (Supplementary Figure 1) and was corrected by incorporating the first two MDS components as covariates in the model for GWAS. A genome-wide scan for significant SNPs considering only additive effects was accomplished through a simple regression model using PLINK v1.9 as described by Marees et al. (2018), where residuals were assumed to be normally and independently distributed. A linear regression model was fitted for determining the association between SNPs and continuous traits (Bush and Moore, 2012), while logistic regression was fitted for the binary trait (lactation persistent/non-persistent based on incomplete gamma function). The threshold for genome-wide significance was determined by correcting the *p-*values of the SNP association test with Benjamini–Hochberg’s false discovery rate (FDR) at 5 and 10% levels (Benjamini and Hochberg, 1995) using the “R” package fuzzySim v3.0 (Barbosa, 2020). A genome-wide significant threshold was set at FDR 5% and a suggestive threshold at 10% by calculating a nominal *p*-value for the largest index **i** for which *P*_(*i*)_ ≤ (**i**/m) × q, where **i** = rank of the SNPs, m = no. of individual tests performed, and q = either 0.05 or 0.1 (Glickman et al., 2014). The results were plotted as Manhattan plots and Q-Q plots using the “qqman” package of R.

***Linear regression model used for GWAS:***


$$y{\text{ = β}}_{\text{0}}\text{ + }x{\text{β}}_{\text{1}}\text{ + }C_{\text{1}}{\text{β}}_{\text{2}}\text{ + }C_{\text{2}}{\text{β}}_{\text{3}}\text{ + }e$$

where *y* = trait, *x* = additive effect of SNPs, C_1_ = first component of MDS, C_2_ = second component of MDS, β_0_ = intercept term, β_1_ = regression coefficient representing the strength of association between SNP *x* and trait *y*, β_2_ = regression coefficient of C_1_, β_3_ = regression coefficient of C_2_, and *e* = residuals or noise not explained by SNPs.

The model used for GWAS on 305DMY, however, included four covariates consisting of the first component of MDS, birth weight, age at first calving, and calving interval.

**TABLE 1:** Details of the top 10 SNPs identified through GWAS on various traits and genes identified through genome scan.

**Trait**	**Chr^†^ no.**	**Position (bp)**	***p*-value**	**Within**	**±20 kb**
PC_1_ of milk yield	X	7,588,358^∗^	1.91 × 10^–07^	*GRIA3*	
	9	62,699,319^∗^	2.39 × 10^–06^	*ZNF292*	
	16	74,362,861^∗^	3.56 × 10^–06^		
	1	182,059,836	1.05 × 10^–05^	Uncharacterized *LOC112444602*	
	6	35,648,660	1.31 × 10^–05^		*TIGD2*
	9	48,559,942	1.38 × 10^–05^	*GRIK2*	
	1	97,587,987	2.68 × 10^–05^	*LRRC34*	*LRRC34*, *ACTRT3*
	20	45,808,430	2.69 × 10^–05^		
	2	72,321,021	3.65 × 10^–05^		
	X	120,205,266	4.13 × 10^–05^		
PC_1_ of fat percentage	6	34,234,112	1.96 × 10^–05^	*CCSER1*	
	20	54,742,629	3.10 × 10^–05^		
	17	15,895,247	5.70 × 10^–05^		
	4	1,579,792	9.68 × 10^–05^		
	16	66,495,537	0.0001022		
	14	27,391,536	0.0001391		
	10	45,942,651	0.0001592		*DAPK2*
	16	46,080,207	0.0001604	*CAMTA1*	
	11	30,281,532	0.0001669		
	4	39,053,011	0.0001739		
PC_1_ of SNF percentage	14	4,664,0635	6.39 × 10^–06^		
	9	104,155,086	6.57 × 10^–06^		
	10	4,420,352	1.03 × 10^–05^		*TICAM2*
	11	88,922,443	1.34 × 10^–05^		
	12	90,113,650	1.64 × 10^–05^		
	3	146,125,164	1.69 × 10^–05^		
	8	99,790,321	2.11 × 10^–05^		*TXN*
	14	38,810,289	2.32 × 10^–05^	*HNF4G*	
	14	54,721,238	2.68 × 10^–05^	*SYBU*	
	17	42,173,707	3.79 × 10^–05^	Uncharacterized *LOC104974614*	
PC_2_ of milk yield	1	81,353,445	4.36 × 10^–06^		
	7	57,678,016	2.86 × 10^–05^	*TCERG1*	
	4	134,421,018	4.95 × 10^–05^		
	16	17,134,516	5.34 × 10^–05^		
	1	49,359,549	7.78 × 10^–05^		
	4	26,233,428	0.0001182		
	13	60,176,598	0.0001579		*ANGPT4*
	18	63,008,459	0.0001706		
	2	85,823,516	0.0001802	*ANKRD44*, uncharacterized *LOC112442949*	
	21	42,799,270	0.0002008		*AKAP6*
PC_2_ of fat percentage	3	81,728,404	9.30 × 10^–05^	*ROR1*	
	15	58,026,658	9.67 × 10^–05^		*CCDC34*
	14	5,954,275	0.0001596		
	6	49,443,269	0.0004445		
	23	25,106,184	0.0004533		*GSTA1*, *GSTA2*
	15	31,296,423	0.0004619	*GRIK4*	
	18	61,661,554	0.0004811	*CACNG6*	
	4	134,458,024	0.0004867		
	7	42,605,598	0.0005285	*SH3BP5L*, *ZNF672*	
	16	74,400,826	0.0005499		
PC_2_ of SNF percentage	14	71,295,596	3.83 × 10^–05^		*TRIQK*
	12	100,102,349	4.23 × 10^–05^		
	1	58,132,034	0.0002283	*CFAP44*	
	20	53,548,085	0.0003363	*CDH18*	
	4	15,355,186	0.0003396		
	6	27,219,584	0.0003841		Uncharacterized *LOC782977*
	22	19,933,711	0.000389		
	5	21,940,540	0.0003954		
	21	56,124,462	0.0004159		
	9	42,153,396	0.0004165	*SEC63*	
305 days’ milk yield	10	5,804,150	2.93 × 10^–05^		
	2	85,890,123	3.90 × 10^–05^		*ANKRD44*
	9	62,699,319	4.52 × 10^–05^	*ZNF292*	
	12	10,144,550	5.91 × 10^–05^		
	X	7,588,358	6.08 × 10^–05^	*GRIA3*	
	16	74,362,861	6.59 × 10^–05^		
	10	5,804,363	8.51 × 10^–05^		
	19	65,955,791	0.0001318		
	1	97,587,987	0.0001527	*MYNN*	*LRRC34*, *ACTRT3*
	9	48,559,942	0.000153	*GRIK2*	
Persistency based on Wood’s function as described by Macciotta	14	2,765,427	0.000693	*DENND3*	
	9	72,904,775	0.000787		
	3	25,896,446	0.001048		
	10	98,483,390	0.001208		
	21	32,145,325	0.001243	*PSTPIP1*	
	5	118,217,420	0.001301		
	19	66,731,726	0.001385		
	6	111,661,570	0.001661		
	1	101,471,188	0.00172		
	5	36,948,158	0.001727	*ADAMTS20*	
Persistency (Mahadevan, 1951)	12	61,269,055	0.0003472		
	18	46,542,221	0.0005129	*PRODH2*, *NPHS1*, *RREL2*, *42466*	
	10	66,128,637	0.000711		
	5	126,657,828	0.0008095		
	14	46,873,549	0.0008564		
	22	33,269,149	0.001113	*FAM19A1*	
	12	72,874,869	0.001206		
	23	19,210,873	0.001381	*CLIC5*	
	12	32,859,855	0.001488	*GPR12*	
	13	60,835,727	0.00154	*DEFB125*	
Persistency (Johansson and Hansson, 1940)	14	45,071,226	1.69 × 10^–05^		
	21	37,776,399	2.76 × 10^–05^		
	9	62,472,303	3.36 × 10^–05^	*CFAP206*	
	18	46,100,792	0.0001026		
	18	46,100,619	0.0001026		
	18	17,868,492	0.0001162	*C18H16orf78*	
	2	20,133,507	0.0001273		
	3	55,760,382	0.0001561		
	4	49,950,395	0.0001709		
	15	11,079,156	0.000183		
Persistency (Ludwick and Petersen, 1943)	6	2,687,0445	6.07 × 10^–05^	*STPG2*	
	9	62,472,303	8.75 × 10^–05^	*CFAP206*	
	18	46,100,792	0.0001368		
	18	46,100,619	0.0001368		
	4	46,668,425	0.0001908	*RINT1*, *EFCAB10*	
	10	65,940,920	0.0002065		
	7	57,678,016	0.0002898	*TCERG1*	
	25	40,090,282	0.0003082		
	10	35,000,168	0.0003138		
	18	17,868,492	0.0003109	*C18H16orf78*	
Breeding efficiency (Tomar, 1965)	10	1,310,267	1.70 × 10^–05^	*APC*, uncharacterized *LOC112448352*	
	11	83,985,064	6.50 × 10^–05^		
	12	54,602,811	0.0003442	*NDFIP2*	
	2	157,056,510	0.0003871		
	X	68,984,061	0.0004459		
	1	40,267,041	0.000448		
	7	117,169,472	0.0005262		
	20	5,188,573	0.0005834	Uncharacterized *LOC107131404*	
	1	182,894,045	0.0006526		
	14	70,378,719	0.0009191		*PDP1*
Age at sexual maturity (in months)	2	19,116,988	2.31 × 10^–05^	*PDE11A*	
	7	77,438,396	5.80 × 10^–05^		
	18	19,894,177	0.0001711		
	3	9,170,745	0.0001738	*SLAMF6*	
	17	58,295,542	0.0001793		uncharacterized *LOC104974658*
	6	32,022,004	0.0001861	*GRID2*	
	1	2,246,590	0.0003256	uncharacterized *LOC104970778*	
	15	75,434,743	0.0003302		
	1	96,574,489	0.0003995	*EIF5A2*, *RPL22L1*	
	10	69,569,504	0.0004181		
Postpartum breeding interval (in days)	2	25,677,863	4.28 × 10^–05^	*ERICH2*	
	4	31,943,618	5.96 × 10^–05^	*IGF2BP3*	*MALSU1*
	14	31,151,162	0.0001547	*PPP1R42*	*TCF24*
	2	69,670,013	0.0002049		*CCDC93*
	6	31,285,865	0.0002096	*GRID2*	
	11	39,293,274	0.0002278		
	7	84,151,709	0.0002888	*EDIL3*	
	7	84,151,714	0.0002947	*EDIL3*	
	13	11,218,416	0.0003107	*LOC112449367*	
	7	17,313,196	0.0003504		Uncharacterized *LOC101904981*, *LOC112447353*

*^†^Chr = chromosome, ^∗^SNPs at the positions were significantly associated with the trait at the 5% level of significance using Benjamini–Hochberg’s FDR.*

### SNP Mapping and Pathway Enrichment

SNPs were identified as genic if present within the genes or intergenic if present within a range of 20 kb from the 5′ and 3′ ends of the gene (da Costa Barros et al., 2018). ARS-UCD1.2/bosTau9 cow assembly was used as the reference genome to identify regions around significant SNPs in the UCSC genome browser (Zimin et al., 2009). Gene ontology (GO) was carried out using gProfiler,^4^ and the GO classifications significant over the Benjamini–Hochberg FDR were selected for pathway enrichment through Cytoscape v3.8.2 (Shannon et al., 2003).

## Results

### PCA on Test-Day Records

PCA on test-day records proved that the first two PCs cumulatively explain 77.72, 40.77, and 48.06% of the total variation in TDMYs, TDFPs, and TDSNFs, respectively. Eigen values of PC_1_ for TDMYs, TDFPs, and TDSNFs were found to be 4.45, 1.67, and 2.30, respectively, while for PC_2_ they were 0.98, 1.20, and 1.05, respectively. New synthetic variable PC_1_ and PC_2_ for the above three traits were constructed for each buffalo utilizing original variables and variable loadings of PCA.

### Genotyping

An average of 1.3 million each of forward and reverse reads were obtained per sample. The total number of forward and reverse end reads was 252.56 million with the average read length being 151 base pairs (bp). After quality check of raw data, it was observed that the average GC content was 50.54%. The rate of duplication was quite high, i.e., 80.11%, which was later checked during variant calling. The quality score (Q) throughout the dataset varied from 35 to 40.

### Variant Calling and Quality Control

Raw files that were quality checked in FastQC were combined using MultiQC, and the report is provided as Supplementary Document. After alignment, BAM files were obtained, and quality was checked. Of the raw reads, 98.11% were mapped accurately to the Mediterranean buffalo reference genome. After removing the duplicate reads, the rate of duplication was reduced to 15.85%. Average mapping quality after alignment was found to be 29.05. A single VCF file was obtained, with 3,854,990 SNPs, out of which 3,792,469 SNPs which were strictly biallelic were retained for further analysis. After applying quality control constraints, 38,560 SNPs present on autosomes and X-chromosomes were retained for further downstream analysis with the final genotyping rate of 98.24%. All the SNPs present on autosome no. 24 failed to pass quality control constraints; hence, in the final analysis, the 24th autosome has been removed.

### GWAS Results

GWAS were performed with 38,560 SNPs, and the genome-wide significant threshold was set at the 5% FDR level. GWAS were performed on the two foremost variation explaining PCs (PC_1_ and PC_2_) of TDMY, TDFP, and TDSNF.

#### GWAS on Lactation Traits

**FIGURE 1:**
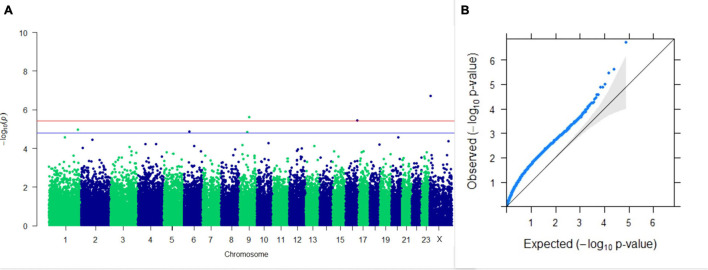
Results of GWAS for PC1 of the test day’s milk yield. **(A)** Manhattan plot of genome-wide SNPs. The red line indicates the *p-*value threshold (expressed as –log_10_*P*) corresponding to FDR-corrected *p-*values or *q* = 0.05, above which the SNPs are considered to be significantly associated with the trait, while the blue line indicates the genome-wide suggestive threshold at *q* = 0.1. The SNPs identified on chromosome no. 24 were screened out from analysis because of stringent quality control; hence, it is not represented in the Manhattan plot. **(B)** Q-Q plot of the *p-*values.

**FIGURE 2:**
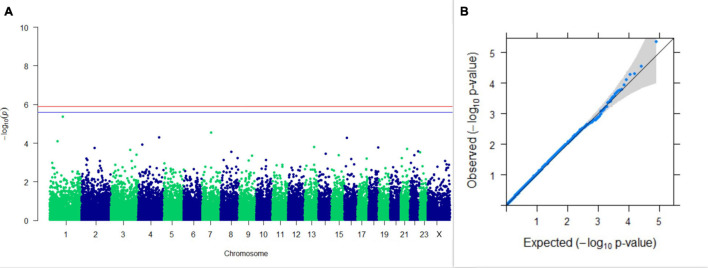
Results of GWAS for PC2 of the test day’s milk yield. **(A)** Manhattan plot of genome-wide SNPs. The red line indicates the *p-*value threshold (expressed as –log_10_*P*) corresponding to FDR-corrected *p-*values or *q* = 0.05, above which the SNPs are considered to be significantly associated with the trait, while the blue line indicates genome-wide suggestive threshold at *q* = 0.1. The SNPs identified on chromosome no. 24 were screened out from analysis because of stringent quality control; hence, it is not represented in the Manhattan plot. **(B)** Q-Q plot of the *p-*values.

**FIGURE 3:**
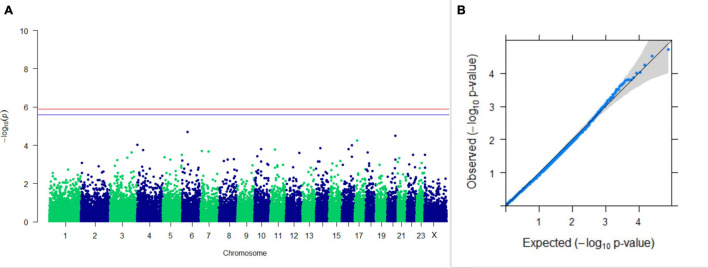
Results of GWAS for PC1 of the test day’s fat percentage. **(A)** Manhattan plot of genome-wide SNPs. The red line indicates the *p-*value threshold (expressed as –log_10_*P*) corresponding to FDR-corrected *p-*values or *q* = 0.05, above which the SNPs are considered to be significantly associated with the trait, while the blue line indicates the genome-wide suggestive threshold at *q* = 0.1. The SNPs identified on chromosome no. 24 were screened out from analysis because of stringent quality control; hence, it is not represented in the Manhattan plot. **(B)** Q-Q plot of the *p-*values.

**FIGURE 4:**
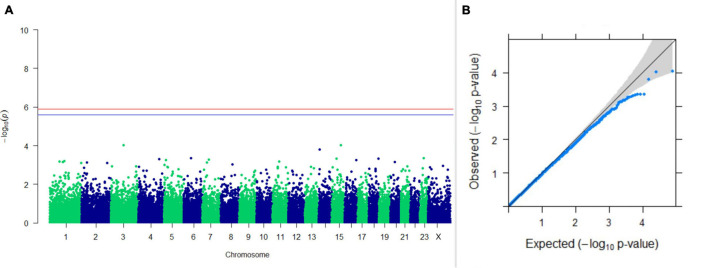
Results of GWAS for PC2 of the test day’s fat percentage. **(A)** Manhattan plot of genome-wide SNPs. The red line indicates the *p-*value threshold (expressed as –log_10_*P*) corresponding to FDR-corrected *p-*values or *q* = 0.05, above which the SNPs are considered to be significantly associated with the trait, while the blue line indicates the genome-wide suggestive threshold at *q* = 0.1. The SNPs identified on chromosome no. 24 were screened out from analysis because of stringent quality control; hence, it is not represented in the Manhattan plot. **(B)** Q-Q plot of the *p-*values.

**FIGURE 5:**
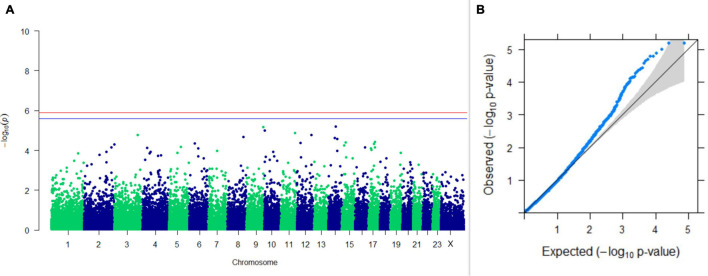
Results of GWAS for PC1 of the test day’s SNF percentage. **(A)** Manhattan plot of genome-wide SNPs. The red line indicates the *p-*value threshold (expressed as –log_10_*P*) corresponding to FDR-corrected *p-*values or *q* = 0.05, above which the SNPs are considered to be significantly associated with the trait, while the blue line indicates the genome-wide suggestive threshold at *q* = 0.1. The SNPs identified on chromosome no. 24 were screened out from analysis because of stringent quality control; hence, it is not represented in the Manhattan plot. **(B)** Q-Q plot of the *p-*values.

**FIGURE 6:**
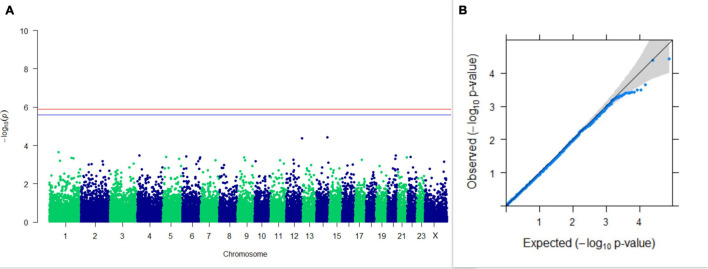
Results of GWAS for PC2 of the test day’s SNF percentage. **(A)** Manhattan plot of genome-wide SNPs. The red line indicates the *p-*value threshold (expressed as –log_10_*P*) corresponding to FDR-corrected *p-*values or *q* = 0.05, above which the SNPs are considered to be significantly associated with the trait, while the blue line indicates the genome-wide suggestive threshold at *q* = 0.1. The SNPs identified on chromosome no. 24 were screened out from analysis because of stringent quality control; hence, it is not represented in the Manhattan plot. **(B)** Q-Q plot of the *p-*values.

Three SNPs present on chromosomes X (7588358 bp), 9 (62699319 bp), and 16 (74362861 bp) were significantly associated with PC_1_ of TDMYs with FDR-corrected *p-*values of 0.007369, 0.04579, and 0.04579, respectively. No SNPs were found to be significantly associated with PC_2_ TDMYs. GWAS on PC_1_ and PC_2_ of TDFPs and TDSNFs also could not establish any significant association between the traits and SNPs. However, the top 10 SNPs having the lowest *p-*values in the test of association with different lactation traits, along with their position and genes within a ± 20-kb region, are listed in Table 1. The Manhattan and Q-Q plots (panels A and B, respectively) for association results of PC_1_–TDMYs, PC_2_–TDMYs, PC_1_–TDFPs, PC_2_–TDFPs, PC_1_–TDSNFs, and PC_2_–TDSNFs are given in Figures 1–6, respectively.

**FIGURE 7:**
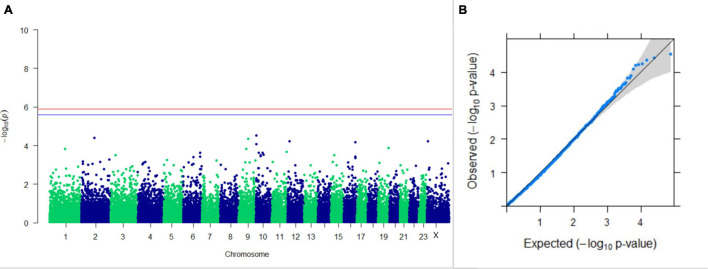
Results of GWAS for 305 days’ milk yield. **(A)** Manhattan plot of genome-wide SNPs. The red line indicates the *p-*value threshold (expressed as –log_10_*P*) corresponding to FDR-corrected *p-*values or *q* = 0.05, above which the SNPs are considered to be significantly associated with the trait, while the blue line indicates the genome-wide suggestive threshold at *q* = 0.1. The SNPs identified on chromosome no. 24 were screened out from analysis because of stringent quality control; hence, it is not represented in the Manhattan plot. **(B)** Q-Q plot of the *p-*values.

GWAS on 305DMY revealed that there was no significant association between SNPs and the trait. It was observed that five SNPs that appeared in the list with GWAS for PC_1_ and PC_2_ of the test day’s milk yield were also in the top SNP list for 305DMY. The Manhattan and Q-Q plots for association results of 305DMY are given in Figures 7A,B, respectively.

#### GWAS on Lactation Persistency

**FIGURE 8:**
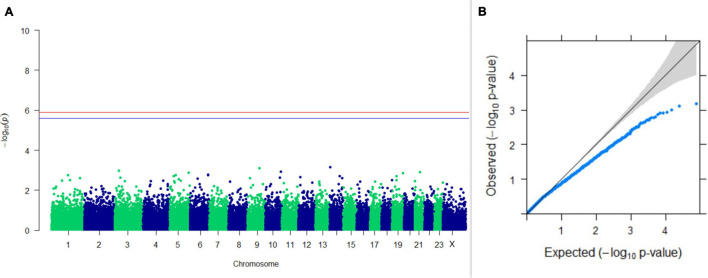
Results of GWAS for lactation persistency based on Wood’s function as described by Macciotta et al. (2005). **(A)** Manhattan plot of genome-wide SNPs. The red line indicates the *p-*value threshold (expressed as –log_10_*P*) corresponding to FDR-corrected *p-*values or *q* = 0.05, above which the SNPs are considered to be significantly associated with the trait, while the blue line indicates the genome-wide suggestive threshold at *q* = 0.1. The SNPs identified on chromosome no. 24 were screened out from analysis because of stringent quality control; hence, it is not represented in the Manhattan plot. **(B)** Q-Q plot of the *p-*values.

**FIGURE 9:**
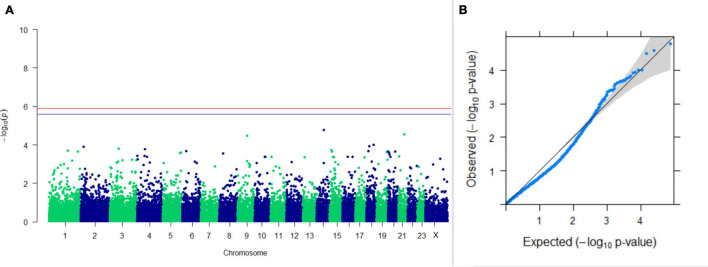
Results of GWAS for lactation persistency according to Johansson and Hansson. **(A)** Manhattan plot of genome-wide SNPs. The red line indicates the *p-*value threshold (expressed as –log_10_*P*) corresponding to FDR-corrected *p-*values or *q* = 0.05, above which the SNPs are considered to be significantly associated with the trait, while the blue line indicates genome-wide the suggestive threshold at *q* = 0.1. The SNPs identified on chromosome no. 24 were screened out from analysis because of stringent quality control; hence, it is not represented in the Manhattan plot. **(B)** Q-Q plot of the *p-*values.

**FIGURE 10:**
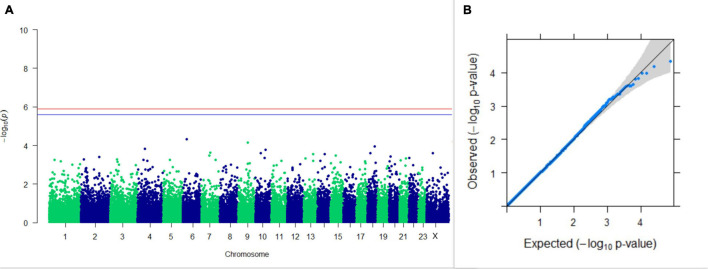
Results of GWAS for lactation persistency according to Ludwick and Peterson. **(A)** Manhattan plot of genome-wide SNPs. The red line indicates the *p-*value threshold (expressed as –log_10_*P*) corresponding to FDR-corrected *p-*values or *q* = 0.05, above which the SNPs are considered to be significantly associated with the trait, while the blue line indicates the genome-wide suggestive threshold at *q* = 0.1. The SNPs identified on chromosome no. 24 were screened out from analysis because of stringent quality control; hence, it is not represented in the Manhattan plot. **(B)** Q-Q plot of the *p-*values.

**FIGURE 11:**
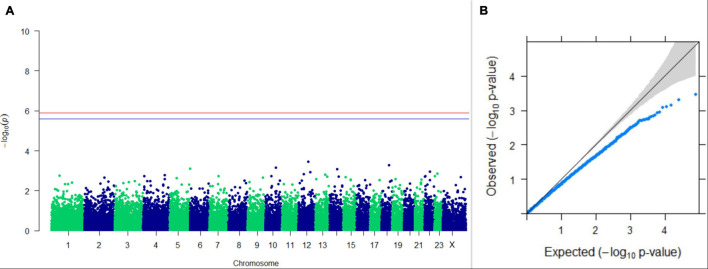
Results of GWAS for lactation persistency according to Mahadevan. **(A)** Manhattan plot of genome-wide SNPs. The red line indicates the *p-*value threshold (expressed as –log_10_*P*) corresponding to FDR-corrected *p-*values or *q* = 0.05, above which the SNPs are considered to be significantly associated with the trait, while the blue line indicates the genome-wide suggestive threshold at *q* = 0.1. The SNPs identified on chromosome no. 24 were screened out from analysis because of stringent quality control; hence, it is not represented in the Manhattan plot. **(B)** Q-Q plot of the *p-*values.

GWAS on lactation persistency estimated by four different methods revealed no significant association between SNPs and the trait generated. The top 10 SNPs having the lowest *p-*values along with their genomic position and genes within a ± 20-kb region are listed in Table 1 for all four methods. It was observed that four SNPs were found in common between the top SNPs listed for persistency estimated by methods II and III. Among the four SNPs, one was on chromosome 9 at 62472303 bp, and three were present on chromosome 18 at 46100792, 46100619, and 17868492 bp. The Manhattan and Q-Q plots (panels A and B, respectively) for association results of persistency estimated by four different methods (I, II, III, and IV) are given in Figures 8–11, respectively.

#### GWAS on Fertility Traits

**FIGURE 12:**
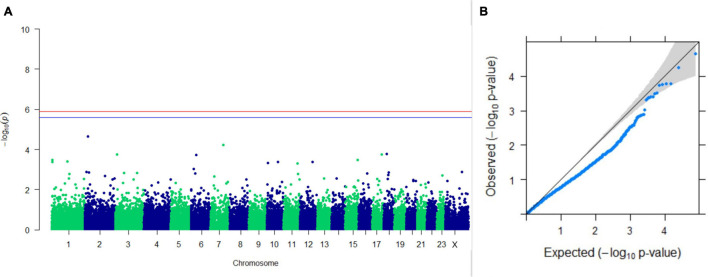
Results of GWAS for age at sexual maturity. **(A)** Manhattan plot of genome-wide SNPs. The red line indicates the *p-*value threshold (expressed as –log_10_*P*) corresponding to FDR-corrected *p-*values or *q* = 0.05, above which the SNPs are considered to be significantly associated with the trait, while the blue line indicates the genome-wide suggestive threshold at *q* = 0.1. The SNPs identified on chromosome no. 24 were screened out from analysis because of stringent quality control; hence, it is not represented in the Manhattan plot. **(B)** Q-Q plot of the *p-*values.

**FIGURE 13:**
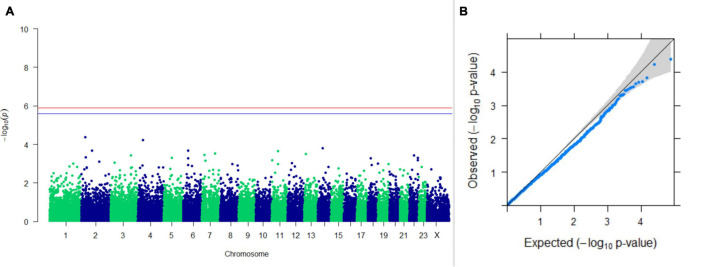
Results of GWAS for postpartum breeding interval. **(A)** Manhattan plot of genome-wide SNPs. The red line indicates the *p-*value threshold (expressed as –log_10_*P*) corresponding to FDR-corrected *p-*values or *q* = 0.05, above which the SNPs are considered to be significantly associated with the trait, while the blue line indicates the genome-wide suggestive threshold at *q* = 0.1. The SNPs identified on chromosome no. 24 were screened out from analysis because of stringent quality control; hence, it is not represented in the Manhattan plot. **(B)** Q-Q plot of the *p-*values.

**FIGURE 14:**
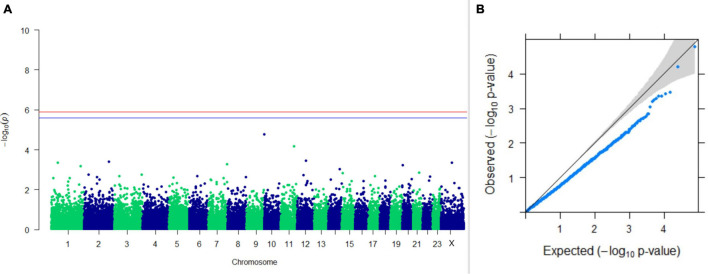
Results of GWAS for breeding efficiency. **(A)** Manhattan plot of genome-wide SNPs. The red line indicates the *p-*value threshold (expressed as –log_10_*P*) corresponding to FDR-corrected *p-*values or *q* = 0.05, above which the SNPs are considered to be significantly associated with the trait, while the blue line indicates the genome-wide suggestive threshold at *q* = 0.1. The SNPs identified on chromosome no. 24 were screened out from analysis because of stringent quality control; hence, it is not represented in the Manhattan plot **(B)** Q-Q plot of the *p-*values.

Upon performing GWAS on age at sexual maturity, postpartum breeding interval, and breeding efficiency, no significant association of any SNPs were observed for the traits; however, the top 10 SNPs with the lowest *p-*values and genes within a ± 20-kb region are listed in Table 1. The Manhattan and Q-Q plots (panels A and B, respectively) of GWAS on age at sexual maturity, postpartum breeding interval, and breeding efficiency are given in Figures 12–14, respectively.

## Discussion

The test-day model (TDM) as repeated measurements of milk yield traits is the method of choice for predicting 305 days’ milk yield. TDMs account for environmental effects on each test day and is useful to model individual lactation curves (Schaeffer et al., 2000; Bignardi et al., 2012). PCA being a powerful multivariate technique is often used to predict 305 days’ milk yield from the inter-related variables such as test-day records (Taggar et al., 2012). Wara et al. (2019) had performed GWAS on test day’s milk yield in Vrindavani cattle; however, reports of GWAS on PCs of test days are very few. In the present study, PCA was applied on seven test-day records of first lactation in order to perform GWAS on the PCs explaining maximum variation rather than the test-day records themselves. The first two PCs cumulatively explained 40–78% of total variation in test-day records of traits included, indicating their potential to be used as GWAS traits to identify novel SNPs for milk yield, fat percentages, and SNF percentages.

It is important to understand the reasons for considering 96 samples taken in the study as optimum, as in the buffalo farming scenario in Asia, particularly in India, the maximum number of buffaloes maintained at any large organized herd ranges from 250 to 500, with 100–200 breedable buffaloes having complete phenotype information. The National Dairy Research Institute has the second-largest herd in the Indian Council of Agricultural Research (ICAR) with a well-managed herd of 250 breedable buffaloes. Furthermore, there is no buffalo sequencing consortium/project in operation (India). In such a case, one could afford this sample size to genotype with complete pedigree and with sufficient genetic diversity. However, the sample size is less for conducting GWAS and raises the question of whether the results are reliable enough. It was observed that the results obtained under the present study are encouraging and important, as one of the genes identified in the present study, i.e., *GRIA3*, was reported by Cole et al. (2011) in a sample of 1,654 US Holstein cows. There are occasions where a similar sample size (96) has been used to understand body morphology traits through GWAS (Rahmatalla et al., 2018).

To identify novel SNPs associated with various economic traits, sequencing through the ddRAD approach was performed. Ruffalo et al. (2012) highlighted that a mapping quality of 15–40 is of the highest nature, and theoretical accuracy corresponds to ∼100%, while depending on the aligner, the actual accuracy of mapping or base call may vary from 40 to 60%. Similar mapping quality averaging 30 for all samples was obtained through BWA aligner, which usually implies good overall base quality of reads and few mismatches in the best possible alignment.

An earlier bovine SNP chip was used to study the traits of buffalo. Using a bovine SNP chip (Illumina BovineSNP50 BeadChip) for a GWAS in buffalo population, Wu et al. (2013) identified seven SNPs that were significantly associated with milk yield. Later on, Affymetrix’s 90K SNP chip commercialization led to several GWAS on milk production traits in buffalo. de Camargo et al. (2015) in a GWAS on buffaloes reported that *LOC100847171*, *BCL6*, *RTP2*, *SST*, *PTGS2*, *LOC100295047*, and *LOC101908004* are associated with milk production; *KCTD8*, *LOC782855*, *LOC101904777*, *ESRRG*, *TRNAY-AUA*, and *GPATCH2* are associated with fat percentage; *LOC101903483*, *SART3*, *ISCU*, *CMKLR1*, *WSCD2*, *MFNG*, *CARD10*, and *USP18* are associated with protein percentage. Liu et al. (2018) identified several candidate genes, namely, *MFSD14A*, *SLC35A3*, *PALMD*, *RGS22*, and *VPS13B*, for milk production in the Italian Mediterranean buffalo through GWAS. In another GWAS in buffalo, Iamartino et al. (2017) reported that genes regulating the D-glucose level in the blood affect milk production in buffalo.

In the present study, GWAS performed on the milk yield PCs explaining maximum variation, which revealed that three SNPs were significantly associated with PC_1_ of TDMY at the 5% FDR level. However, six SNPs present on chromosomes 1, 6, and 9 at 182,059,836, 35,648,660, and 48,559,942 bp, respectively, were above the suggestive threshold of 10% FDR and are presented in Table 1 as the top six SNPs associated with PC_1_ of TDMY. However, for the rest of the traits studied, no SNPs were detected to be significantly associated (or as rejections) at 5% or even 10% FDR.

Upon scanning ± 20 kb around that SNP on the X chromosome, the *GRIA3* gene was found. Glutamate ionotropic receptor AMPA type subunit 3 (*GRIA3*) is reported to be very significantly associated with daughter pregnancy rate in US Holstein cows (Cole et al., 2011). In a study, a positive selection signal has been observed for the loci containing the *CNIH3* gene. *CNIH3* interacts with *GRIA1*, *GRIA2*, *GRIA3*, *GRIA4*, and *GRIK1* belonging to a class of α-amino-3-hydroxy-5-methyl-4-isoxazolepropionic acid (AMPA) glutamate receptors, thus regulating trafficking of AMPA receptors. AMPA receptors are known to participate in luteinizing hormone (LH) secretion (Utsunomiya et al., 2013). The nearest genes to the top 10 SNPs from PC1 GWAS for milk yield were *ZNF292*, *LOC112444602*, *TIGD2*, *GRIK2*, and *LRRC34*. Similarly, for PC2 of milk yield, GWAS revealed that *TCERG1*, *ANGPT4*, *ANKRD44*, *LOC112442949*, and *AKAP6* were the nearest genes from the top 10 SNPs. It could be observed that five SNPs present in the list of top SNPs from GWAS results of PC_1_ and PC_2_ of the test day’s milk yield were also present in the top 10 SNP list for 305 days’ milk yield (Table 1), highlighting the efficacy of PCA in predicting 305 days’ milk yield. Additionally, *MYNN* and *ACTRT3* were found nearest to the top SNPs of GWAS for 305 days’ milk yield. Cai et al. (2020) have also reported the *ANKRD44* gene to be associated with milk yield in Nordic Holstein cattle.

A genome-wide scan for novel genes for fat percentage outlined *CCSER1*, *DAPK2*, *CAMTA1*, *ROR1*, *CCDC34*, *GSTA1*, *GSTA2*, *GRIK4*, *CACNG6*, *SH3BP5L*, and *ZNF672* as the nearest genes to the top SNPs obtained from GWAS. Kolbehdari et al. (2009) in a whole-genome study in Canadian Holstein cattle mapped the gene *DAPK2* to several milk production-related QTLs. They also mapped *DAPK2* to a milk fat percentage QTL which concords with the findings of our study. Genes other than *DAPK2* were not reported earlier to be associated with milk fat percentage in buffalo; however, *CAMTA1* may possibly have a role in fatty acid metabolism.

*TICAM2*, *TXN*, *HNF4G*, *SYBU*, *LOC104974614*, *TRIQK*, *CFAP44*, *CDH18*, *LOC782977*, and *SEC63* were found near the top SNPs associated with PCs of TDSNF. We could not find any previous reports stating the role of these genes in regulating milk SNF percentage; however, a functional enrichment analysis study by Zhou et al. (2019) revealed *HNF4G* and *SYBU* as candidate genes that regulate milk fat synthesis, transport, and metabolism. All the genes involved with milk yield and its composition identified through the study were enriched for pathways in Cytoscape. A sub-network of the genes is depicted in Supplementary Figure 2. It can be observed that most of the genes identified belong to the glutamate receptor family, involved in the regulation of the neuronal system.

In Canadian Holstein cattle, Do et al. (2017) reported a significant genetic effect of *MAN1C1*, *MAP3K5*, *HCN1*, *TSPAN9*, *MRPS30*, *TEX14*, and *CCL28* genes on lactation persistency. Deng et al. (2019) reported that the *MAP3K5* gene regulates p38 MAPKs and Jun N-terminal kinases (JNKs) pathways involved in mammary gland development. In the present study, lactation persistency was calculated using four different methods, and it was observed that the top four SNPs identified by GWAS for persistency as estimated by the Johansson and Hansson (1940) method were in common with the GWAS results obtained for persistency as per the Ludwick and Petersen (1943) method. The genes present near the top SNPs were *DENND3*, *PSTPIP1*, *ADAMTS20*, *PRODH2*, *NPHS1*, *RREL2*, *FAM19A1*, *CLIC5*, *GPR12*, *DEFB125*, *STPG2*, *CFAP206*, *RINT1*, *EFCAB10*, *TCERG1*, and *C18H16orf78*. Upon network enrichment, it was observed that *TCERG1*, *RINT1*, *CLIC5*, and *PSTPIP1* are co-expressed with several other genes in the breast mammary tissue of human, indicating their possible role in persistency of lactation in dairy animals.

GWAS for fertility traits were performed on breeding efficiency, age at sexual maturity, and postpartum breeding interval. Genes present near the top SNPs of breeding efficiency were *APC*, *LOC112448352*, *NDFIP2*, *LOC107131404*, and *PDP1*. Mohamed et al. (2019) reported that *APC* gene in the canonical WNT signaling pathway plays a critical role in the regulation of ovarian development. Mis-regulation of this key pathway in the adult ovary is associated with subfertility in mice. The *APC* gene has also been mapped to the QTL region for conception rate in Holstein cattle (Kolbehdari et al., 2009). Genes identified near the top SNPs for age at sexual maturity are *PDE11A*, *SLAMF6*, *LOC104974658*, *GRID2*, *LOC104970778*, *EIF5A2*, and *RPL22L1*. Coyral-Castel et al. (2018) mapped the *SLAMF6* gene to the QTL region affecting female fertility located on the bovine chromosome three (QTL-F-Fert-BTA3). The *GRID2* gene is however reported to be associated with growth traits in Simmental cattle and may have role in regulating age at sexual maturity (Braz et al., 2020). Christensen et al. (2005) have reported *EIF5A2* as a candidate gene for infertility in human. Genes identified for postpartum breeding interval were *ERICH2*, *MALSU1*, *IGF2BP3*, *TCF24*, *PPP1R42*, *CCDC93*, *GRID2*, *EDIL3*, *LOC112449367*, *LOC101904981*, and *LOC112447353*. However, none of these genes was identified to be involved significantly in any pathway through the gProfiler.

Although we have already emphasized that this a preliminary GWAS on buffaloes covering a large set of economic traits (lactation, lactation persistency, and fertility), the results obtained in the present study are bias free, as indicated by the FDR. The findings of the present study in the Murrah population will encourage researchers to come forward for GWAS in buffalo in a holistic manner.

## Conclusion

Optimum production and reproduction in buffaloes are long-standing questions in the Indian subcontinent. We present the analysis and identification of genomic regions that play role in shaping the selection and breeding decisions of Murrah buffalo for persistency of production and fertility. The dataset information presented in the paper is also submitted so that it can be used to compare and evaluate other breeds of buffalo based on the genomic information generated. The putative identified regions have a potential to improve the existing breeding decisions in this important dairy germplasm.

## Data Availability Statement

The data presented in the study are deposited in the European Variation Archive repository, accession number PRJEB47270 (https://wwwdev.ebi.ac.uk/eva/?eva-study=PRJEB47270).

## Ethics Statement

The animal study was reviewed and approved by the ICAR-National Dairy Research Institute IAEC.

## Author Contributions

VV conceptualized the work and interpreted the data. SC, GG, RA, and AM performed the collection of materials, and performed the data analysis. AV and SD provided the data and resources. VV and SC did writing of the manuscript and its critical evaluation. All authors contributed to the article and approved the submitted version.

## Conflict of Interest

The authors declare that the research was conducted in the absence of any commercial or financial relationships that could be construed as a potential conflict of interest.

## Publisher’s Note

All claims expressed in this article are solely those of the authors and do not necessarily represent those of their affiliated organizations, or those of the publisher, the editors and the reviewers. Any product that may be evaluated in this article, or claim that may be made by its manufacturer, is not guaranteed or endorsed by the publisher.

## Footnotes

1
https://dahd.nic.in/about-us/divisions/statistics

2
https://github.com/broadinstitute/picard

3
https://sourceforge.net/projects/bbmap/

4
https://biit.cs.ut.ee/gprofiler/gost

## Supplementary Material

Supplementary Figure 1 MDS plot based on the first and second MDS components showing population stratification.

Supplementary Figure 2 Sub-network of enriched pathways of genes identified responsible for milk yield and its composition.
