# Supplementary figures and images for: Genome-Wide Association Studies in Indian Buffalo Revealed Genomic Regions for Lactation and Fertility
Source: Front Genet. 2021 Sep 20;12:696109. doi: 10.3389/fgene.2021.696109 (PMC8488374; doi:10.3389/fgene.2021.696109)

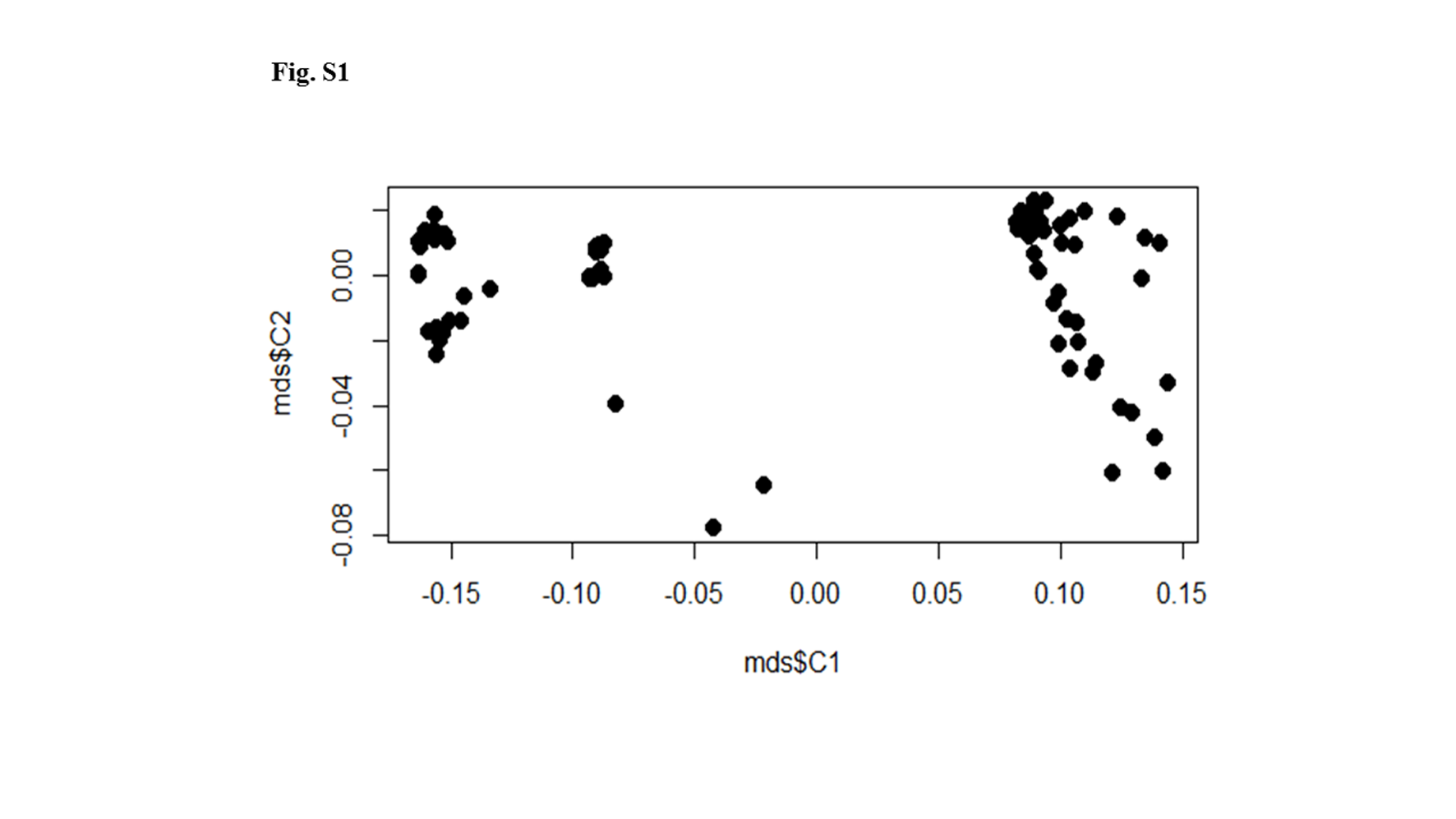

Supplement: Supplementary Figure 1 — MDS plot based on the first and second MDS components showing population stratification. [file Image_5.TIF]

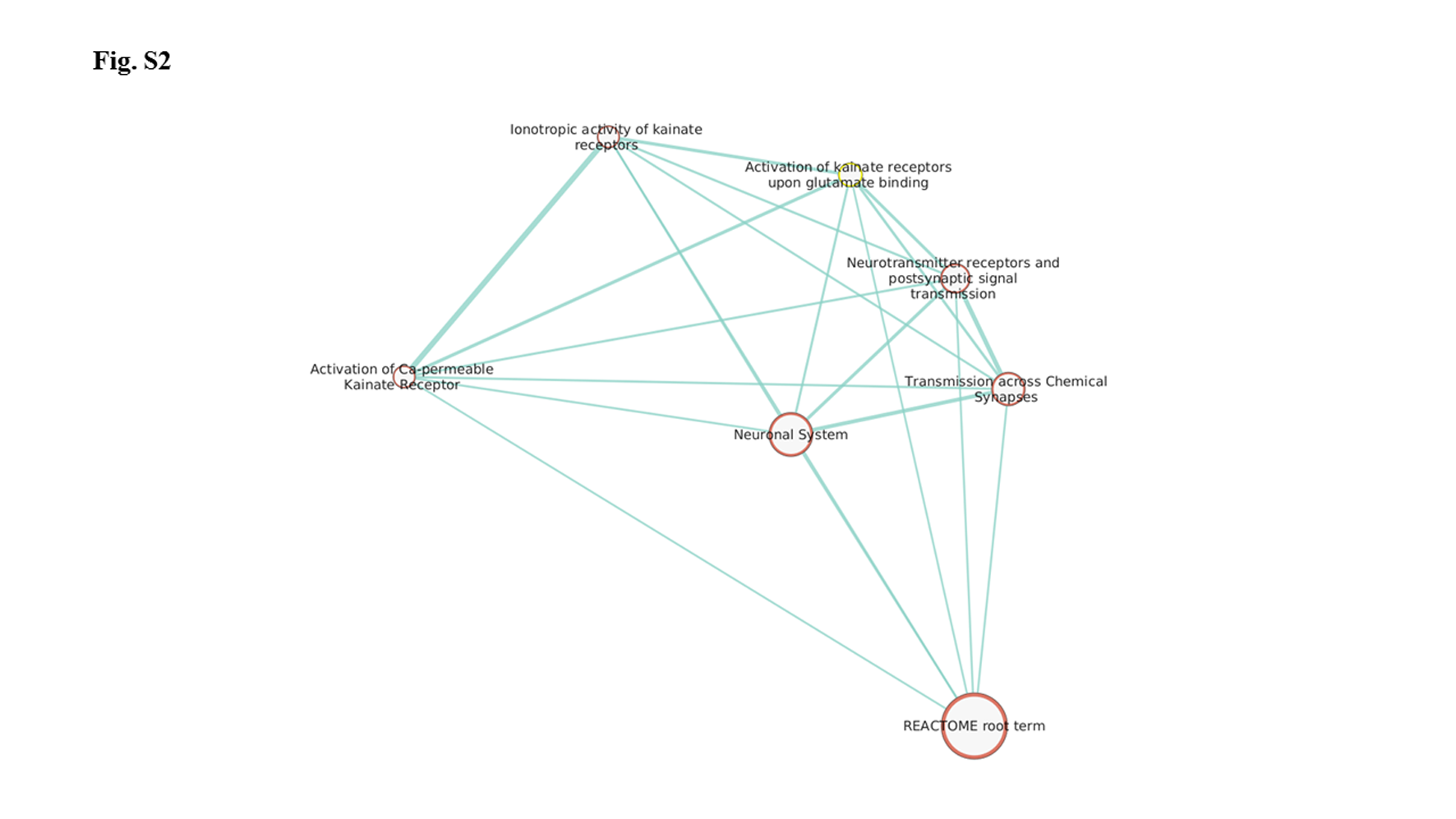

Supplement: Supplementary Figure 2 — Sub-network of enriched pathways of genes identified responsible for milk yield and its composition. [file Image_6.TIF]

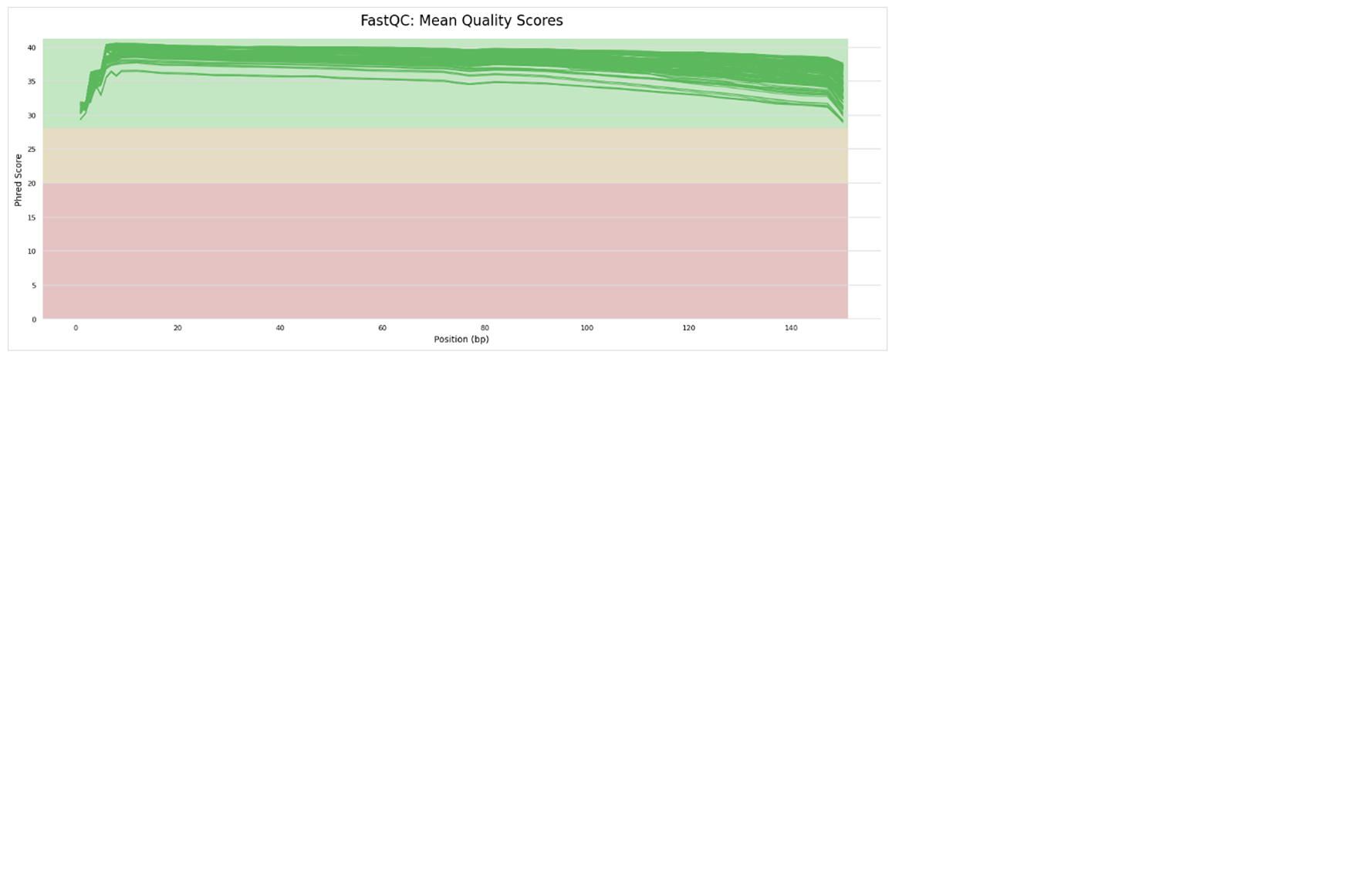

Supplement: Supplementary file 3 [file Image_1.PNG]

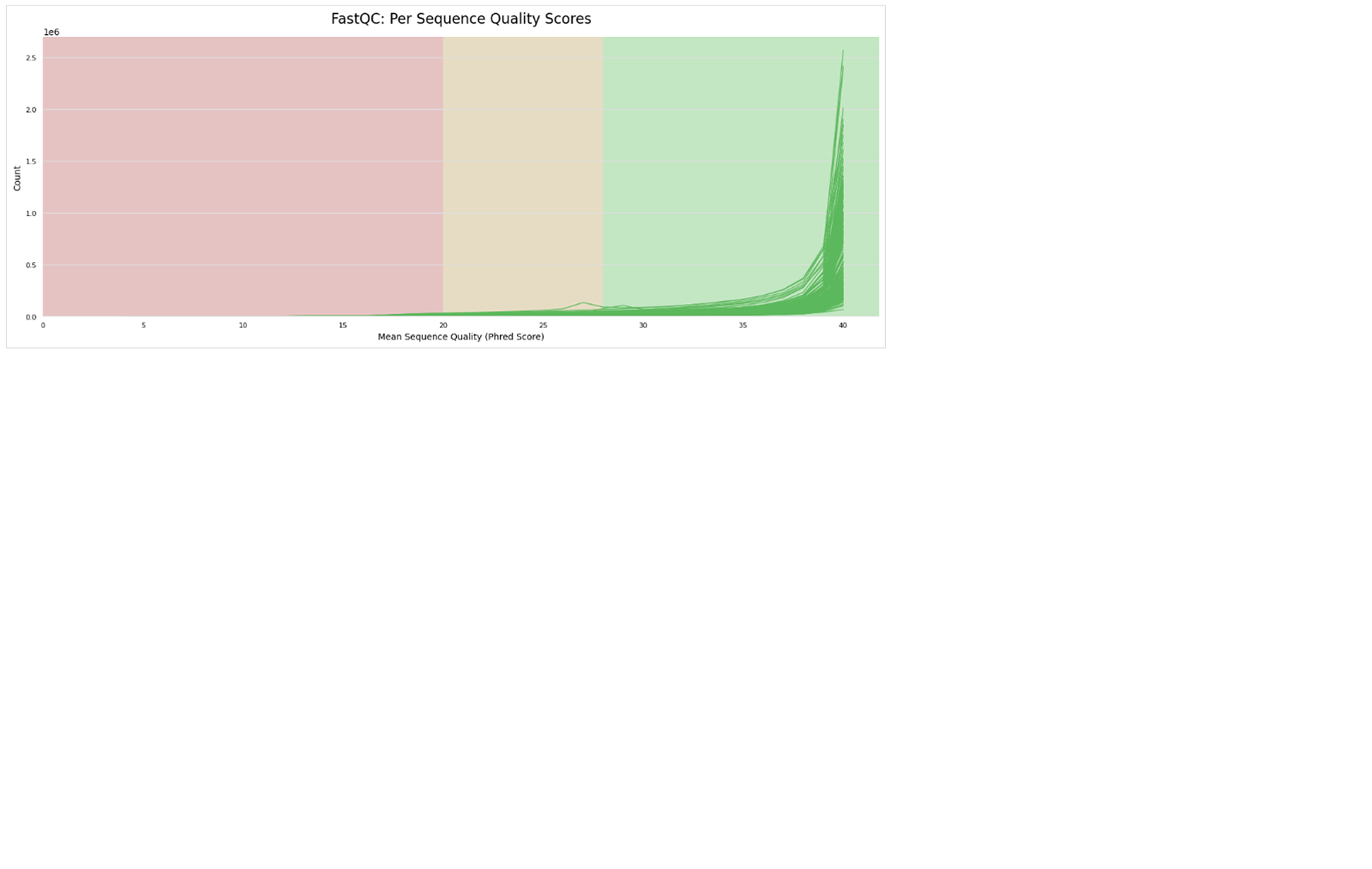

Supplement: Supplementary file 4 [file Image_2.PNG]

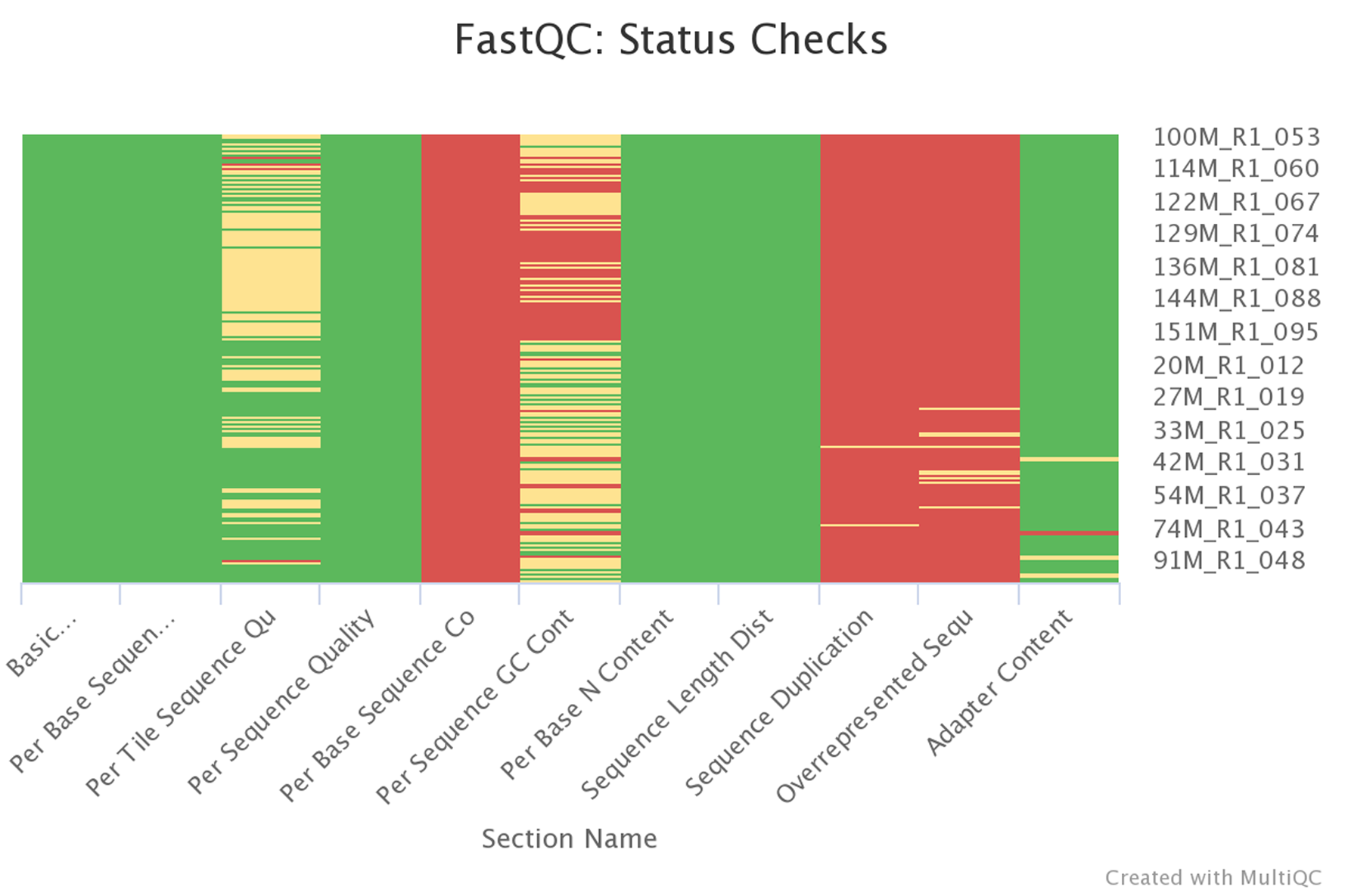

Supplement: Supplementary file 5 [file Image_3.PNG]

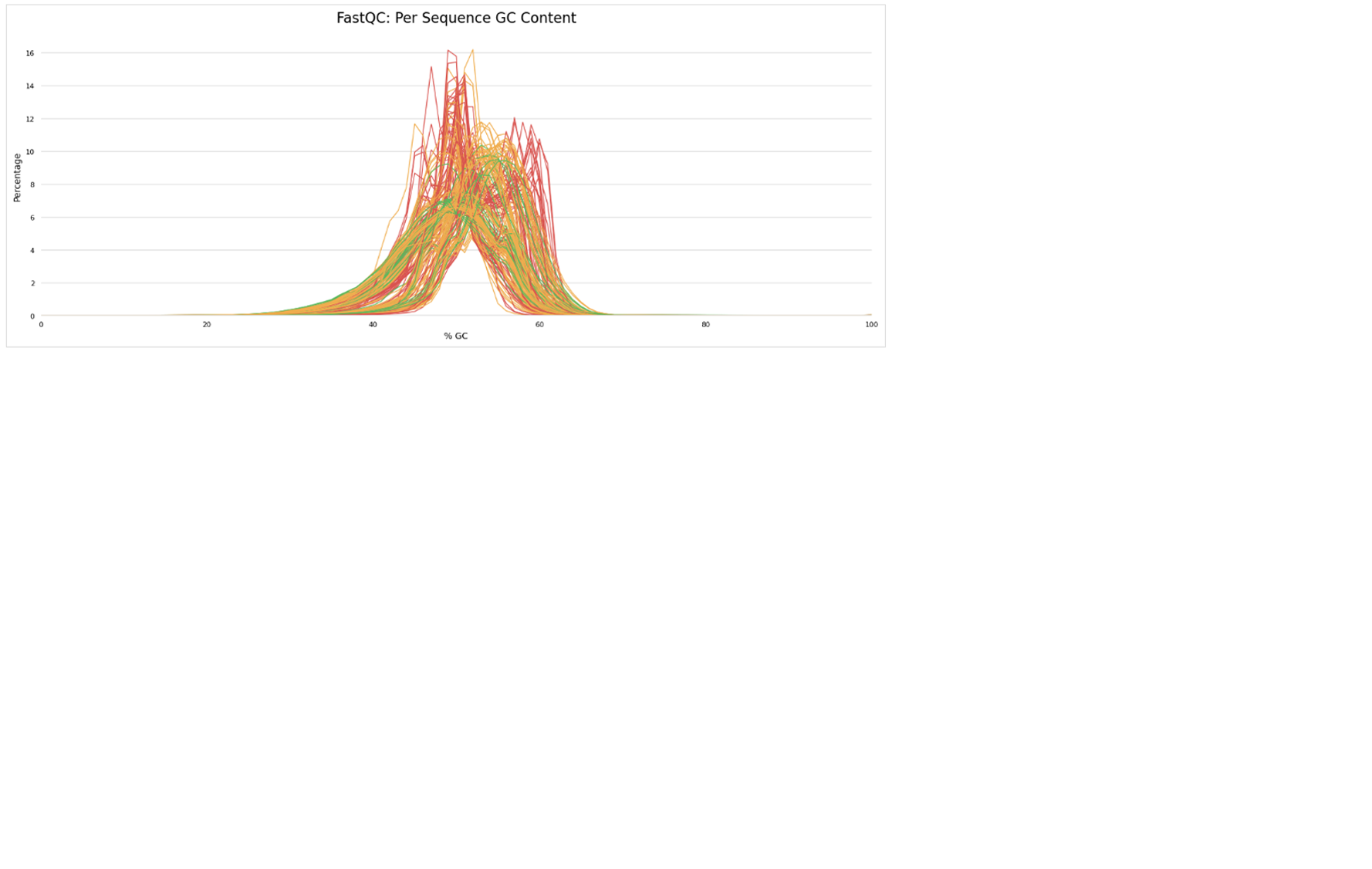

Supplement: Supplementary file 6 [file Image_4.PNG]
